# Supplementary material for: Post-Operative Anorectal Manometry in Children following Anorectal Malformation Repair: A Systematic Review
Source: J Clin Med. 2023 Mar 28;12(7):2543. doi: 10.3390/jcm12072543 (PMC10094903; doi:10.3390/jcm12072543)
Supplement: Supplementary file 1 [file jcm-12-02543-s001.zip › jcm-2215573-supplementary.pdf]

SUPPLEMENTARY MATERIAL

Table S1: The Newcastle-Ottawa Scale: study quality assessment [11]

Criteria	Arnoldi [14]	Banasiuk [64]	Becmeur [23]	Bhat [56]	Burjonrappa [61]	Cahill [24]	Caldaro [43]	Caruso [67]	Caruso [72]	Chen [73]	Chung [22]	Doolin [44]	El-Debeiky [25]
**Selection**[1]													
1. Representativeness of the exposed cohort	-	★	-	★	-	-	★	★	★	★	★	★	★
2. Selection of the non-exposed cohort	★	-	-	-	★	-	-	-	-	-	★	-	-
3. Ascertainment of exposure	★	★	★	★	-	★	★	★	★	★	★	★	★
4. Demonstration that outcome of interest was not present at start of study	★	★	★	★	★	★	★	★	★	★	★	★	★
**Comparability**[2]													
1. Comparability of cohorts on the basis of the design or analysis	★	-	-	★	-	-	★	-	-	-	★	-	-
**Outcome**[3]													
1. Assessment of outcome	★	-	-	-	★	-	-	-	-	-	★	-	-
2. Was follow-up long enough for outcomes to occur	★	-	★	-	★	-	★	★	★	★	★	★	-
3. Adequacy of follow up of cohorts	★	★	★	★	★	★	★	★	★	★	★	★	★
**Assessment**	*Good*	*Poor*	*Poor*	*Poor*	*Poor*	*Poor*	*Good*	*Poor*	*Poor*	*Poor*	*Good*	*Poor*	*Poor*

[1] **Selection**: (1) *Representativeness of the exposed cohort*: A, truly representative ★; B, somewhat representative ★; C, selected group; and D, no description of the derivation of the cohort. (2*) Selection of the nonexposed cohort*: A, drawn from the same community as the exposed cohort ★; B, drawn from a different source; and C, no description of the derivation of the non-exposed cohort. (3) *Ascertainment of exposure*: A, secure record (e.g., surgical records) ★; B, structured interview ★; C, written self-report; D, no description, and E, other. (4) *Demonstration that outcome of interest was not present at start of study*: A, yes ★; B, no.

[2] **Comparability**: *Comparability of cohorts on the basis of the design or analysis*: A, study controls for age ★; B, study controls for other factors (such as treatment type) ★; and C, cohorts are not comparable on the basis of the design or analysis controlled for confounders.

[3] **Outcome**: (1) *Assessment of outcome*: A, independent blind assessment ★; B, record linkage ★; C, self-report; D, no description, and E, other. (2) *Was follow-up long enough for outcomes to occur*: A, yes ★; B, no. (3) *Adequacy of follow up of cohorts*: A, complete follow-up - all subjects accounted for ★; B, subjects lost to follow up unlikely to introduce bias (loss ≤20%) or description provided of those lost no different from those followed ★; C, follow up rate <80% and no description of those lost; and D, no statement.

Criteria	Emblem [40]	Emblem [45]	Fukata [41]	Hedlund [46]	Heikenen [74]	Hettiarachc hi [47]	Huang [20]	Husberg [26]	Ishihara [48]	Iwai [65]	Iwai [66]	Iwai [68]
**Selection**												
1. Representativeness of the exposed cohort	★	-	-	★	-	-	★	★	★	★	-	★
2. Selection of the non-exposed cohort	★	-	-	-	-	-	★	-	-	-	-	-
3. Ascertainment of exposure	★	★	★	★	★	★	★	★	★	★	-	★
4. Demonstration that outcome of interest was not present at start of study	★	★	★	★	★	★	★	★	★	★	★	★
**Comparability**												
1. Comparability of cohorts on the basis of the design or analysis	★	★	-	-	-	-	-	-	★	-	-	-
**Outcome**												
1. Assessment of outcome	-	-	-	-	-	★	-	-	-	-	-	-
2. Was follow-up long enough for outcomes to occur	★	★	★	★	★	-	★	★	-	-	-	-
3. Adequacy of follow up of cohorts	★	★	★	★	★	-	-	★	★	★	★	★
**Assessment**	*Good*	*Fair*	*Poor*	*Poor*	*Poor*	*Poor*	*Poor*	*Poor*	*Poor*	*Poor*	*Poor*	*Poor*

Criteria	Iwai [15]	Keshtgar [69]	Keshtgar [49]	Kimura [27]	Kudou [28]	Kumar [57]	Langemeijer [29]	Leung [70]	Lin [30]	Lin [31]	Liu [32]	Martins [50]	Mert [55]
**Selection**													
1. Representativeness of the exposed cohort	-	★	★	★	★	★	★	★	★	★	★	★	★
2. Selection of the non-exposed cohort	-	-	★	★	-	★	-	-	-	★	★	-	-
3. Ascertainment of exposure	★	★	★	★	★	★	★	★	★	★	★	-	★
4. Demonstration that outcome of interest was not present at start of study	★	★	★	★	★	★	★	★	★	★	★	★	★
**Comparability**													
1. Comparability of cohorts on the basis of the design or analysis	-	-	-	-	-	★	-	-	-	★	-	★	-
**Outcome**													
1. Assessment of outcome	-	-	★	★	-	★	-	-	★	-	★	-	-
2. Was follow-up long enough for outcomes to occur	★	★	-	★	★	-	★	★	-	★	★	-	-
3. Adequacy of follow up of cohorts	★	★	★	★	★	★	-	★	★	★	★	★	★
**Assessment**	*Poor*	*Poor*	*Poor*	*Poor*	*Poor*	*Good*	*Poor*	*Poor*	*Poor*	*Good*	*Poor*	*Poor*	*Poor*

Criteria	Mollard [75]	Nagashima [62]	Niedzielski[33]	Ninan [71]	Okada [21]	Penninckx [16]	Ray [76]	Ren [34]	Rintala [35]	Rintala [36]	Rintala [58]	Rintala [17]
**Selection**												
1. Representativeness of the exposed cohort	-	★	★	★	-	-	★	★	-	★	★	-
2. Selection of the non-exposed cohort	★	-	★	-	-	-	-	★	-	★	★	-
3. Ascertainment of exposure	★	★	★	★	★	★	★	★	★	★	★	★
4. Demonstration that outcome of interest was not present at start of study	★	★	★	★	★	★	★	★	★	★	★	★
**Comparability**												
1. Comparability of cohorts on the basis of the design or analysis	-	-	★	-	-	-	-	★	-	-	★	-
**Outcome**												
1. Assessment of outcome	★	-	★	-	★	-	-	★	★	-	-	-
2. Was follow-up long enough for outcomes to occur	★	-	★	★	★	-	-	★	★	-	★	-
3. Adequacy of follow up of cohorts	-	★	★	★	★	-	★	-	★	★	★	★
**Assessment**	*Poor*	*Poor*	*Good*	*Poor*	*Poor*	*Poor*	*Poor*	*Good*	*Poor*	*Poor*	*Good*	*Poor*

Criteria	Rintala [59]	Rintala [60]	Ruttenstock [18]	Sangkhathat [63]	Schuster [19]	Schuster [42]	Senel [51]	Sonnino [37]	Tang [52]	Tong [38]
**Selection**										
1. Representativeness of the exposed cohort	★	-	★	-	★	-	★	-	-	★
2. Selection of the non-exposed cohort	★	-	-	-	★	★	-	-	★	★
3. Ascertainment of exposure	★	★	★	★	★	★	★	★	★	★
4. Demonstration that outcome of interest was not present at start of study	★	★	★	★	★	★	★	★	★	★
**Comparability**										
1. Comparability of cohorts on the basis of the design or analysis	★★	-	★	-	★	★	-	-	-	-
**Outcome**										
1. Assessment of outcome	★	-	-	-	-	★	-	-	★	★
2. Was follow-up long enough for outcomes to occur	★	★	-	-	-	★	★	-	-	★
3. Adequacy of follow up of cohorts	★	★	★	★	★	★	★	★	★	★
**Assessment**	*Good*	*Poor*	*Poor*	*Poor*	*Poor*	*Good*	*Poor*	*Poor*	*Poor*	*Poor*

Criteria	Vital Junior [53]	Wang [54]	Yang [39]
**Selection**			
1. Representativeness of the exposed cohort	★	★	★
2. Selection of the non-exposed cohort	-	★	-
3. Ascertainment of exposure	★	★	★
4. Demonstration that outcome of interest was not present at start of study	★	★	★
**Comparability**			
1. Comparability of cohorts on the basis of the design or analysis	-	-	★
**Outcome**			
1. Assessment of outcome	★	★	★
2. Was follow-up long enough for outcomes to occur	-	★	-
3. Adequacy of follow up of cohorts	★	★	★
**Assessment**	*Poor*	*Poor*	*Good*

Table S2: Summary of consistently reported anorectal manometry parameters: parameter definitions, resting pressure, squeeze pressure, and rectoanal inhibitory reflex. Units of pressure standardized to mmHg.

First author	Resting pressure	Squeeze pressure	RAIR	Resting pressure (mmHg)	Squeeze pressure *(mmHg)*	RAIR (% present)
**Arnoldi** [14]	**Anal resting pressure:** the mean pressure that was registered during the passage of the catheter through the anal canal.	-	**Rectoanal inhibitory reflex**: anal pressure decrease of >5mmHg following balloon inflation of 10 – 50mL.	**Fistula location:**<br>*Rectoperineal:* 31 (15 – 40).<br>*Rectovestibular:* 29.5 (15 – 45).<br>**Neurospinal dysraphism**<br>*Present:* 23 (15 – 35).<br>*Absent:* 32 (15 – 45).<br>**Neonatal colostomy**<br>*Yes*: 21 (15 – 45).<br>*No*: 32 (20 – 40).	-	77%
**Banasiuk** [64]	**Resting pressure**: not further defined.	**Squeeze pressure:** performed twice. Not further defined.	**Rectoanal inhibitory reflex:** 25% decrease in mean resting pressure.	Median 56.6 (5th – 95th centiles: 12.7 – 82.8)	Median 121.7 (5th – 95th centiles: 38.2 – 46.8)	50%
**Becmeur** [23]	*Normal b*asal resting pressure: >29.5mmHg.	-	*Normal*: presence of **rectoanal relaxation reflex** at 7.35mmHg.	-	-	-
**Bhat** [56]	Mean anal canal pressure of the neo-anorectum.	-	-	Post-PSARP: 6.1 – 68.6<br>Post colostomy closure: 16.7 – 72.9	-	-
**Burjonrappa** [61]	-	-	**Anorectal inhibitory reflex**: elicitation by distension of rectum with air. Not further defined.	-	-	83%
**Cahill** [24]	**Mean anal canal pressure**: pressure between 25 and 35mmHg in the healthy child.	-	**Anal inhibitory reflex:** relaxation of the internal sphincter when distended with a balloon.	Median canal pressure: 36 (16 – 40)	-	100%

First author	Resting pressure	Squeeze pressure	RAIR	Resting pressure (mmHg)	Squeeze pressure *(mmHg)*	RAIR (% present)
**Caldaro** [43]	Average anal resting pressure (aARP).<br>Contributes to anorectal manometry scoring system[4]:<br><20mmHg: 2<br>>20mmHg: 0	Average anal squeeze pressure (aASP).<br>Contributes to anorectal manometry scoring system:<br><55mmHg: 2<br>>55mmHg: 0	Rectoanal inhibitory reflex (RAIR).<br>Contributes to anorectal manometry scoring system:<br>Absent: 2<br>Present: 0	Group 1 (low): 42 ± 11<br>Group 2 (intermediate): 20±6<br>Group 3 (high): 14±9	Group 1 (low): 57 ± 43<br>Group 2 (intermediate): 46±28<br>Group 3 (high): 24±14	Group 1 (low): 83%<br>Group 2 (intermediate): 50%<br>Group 3 (high): 16.7%
**Caruso** [67]	Average resting pressure.<br>>20mmHg: favorable<br><20mmHg: unfavorable	**Average anal squeeze pressure**: increment of average RP with maximal voluntary contraction.<br>>20mmHg: favorable<br><20mmHg: unfavorable	-	Pre-treatment:<br>Group 1: 26.3 ± 4.5<br>Group 2: 25.5 ± 4.9<br>Group 3: 17.5 ± 2.1<br>Group 4: 12.7 ± 2.2<br>Post-treatment:<br>Group 1: 51.7 ± 6.5<br>Group 2: 44.0 ± 4.7<br>Group 3: 36.3 ± 8.7<br>Group 4: 18.7 ± 4.9	Pre-treatment:<br>Group 1: 32.8 ± 3.6<br>Group 2: 23.6 ± 3.2<br>Group 3: 16.6 ± 2.0<br>Group 4: 6.0 ± 4.3<br>Post-treatment:<br>Group 1: 50.4 ± 5.4<br>Group 2: 39.6 ± 7.5<br>Group 3: 22.6 ± 4.5<br>Group 4: 14.5 ± 4.4	-
**Caruso** [72]	**Mean resting anal pressure**: not further defined.	**Maximum voluntary contraction pressure during squeeze:** reported as increment versus resting pressure.	-	36.14 ± 12.94	34.29 ± 8.3	-
**Chen** [73]	**Resting anal pressure:** measured in anal canal for 30s; anorectal profile calculated as the catheter was pulled through at 5mm/min.<br>**Resting rectal pressure:** measured for 30s at 5-10cm above the anal verge, according to the patient's age [30].	-	**Rectoanal reflex:** positive reflex: pressure decrease of more than half the resting anal pressure, after inflation of the balloon in 5mL increments, 5-10cm from the anal verge [30].	**<2 years**<br>*LSARP*: 31.3 ± 7.4 (constipated); 24.8 ± 4.0 (not constipated).<br>*PSARP*: 29.9 ± 4.5 (constipated); 22.4 ± 5.1 (not constipated).<br>*R-ASPA*: 30.1 ± 7.4<br>**>2 years**<br>*LSARP* + *PSARP*: 27.5 ± 5.5 (constipated); 24.0 ± 5.6 (not constipated); 18.1 ± 4.8 (soiling).<br>*R-ASPA*: 27.4 ± 11.3	-	33.3% - 91.7%
**Chung** [22]	Sphincteric resting pressure: "normal" 30 – 60mmHg.	Squeeze pressure: "normal" 50 – 120mmHg.	Rectoanal inhibitory reflex (RAIR): considered positive if >15mmHg decrease in resting pressure for 5s.	PSARP: median 20 mmHg (range 10 – 50 mmHg).<br>LARP: median 40 mmHg (range 10 – 70 mmHg).	PSARP: median 30 mmHg (range 10 – 100 mmHg).<br>LARP: median 50 mmHg (range 20 – 140 mmHg).	PSARP: 42.9%<br>LARP: 31.3%

[4] Anorectal manometry scoring system devised to determine activity of IAS and EAS: scores of 0 and 1 indicate good, and scores of 2 to 5, poor activity.

First author	Resting pressure	Squeeze pressure	RAIR	Resting pressure (mmHg)	Squeeze pressure *(mmHg)*	RAIR (% present)
**Doolin** [44]	**Baseline rectal pressure**: pressure when the patient was at rest.	**Voluntary contraction pressure**: pressure that could be generated above baseline pressure on command.	**Relaxation reflex**: decrease of anal pressure in response to rectal dilatation. Defined as yes, no, or paradoxical (increase of baseline rectal tone when balloon was inflated).	Mean 14.7 (range 0 – 33.1)	Mean 11.0 (range 0 – 72.8)	23%
**El-Debeiky** [25]	**Resting pressure.** Considered abnormal if "no measurements of the resting pressure". Not further defined.	**Squeezing pressure:** measured by asking the child to withhold urine to stimulate voluntary contractions without the aid of the buttocks. Considered abnormal if "failure of pressure increasing with maximum squeezing".	**Rectoanal inhibitory reflex**: evaluated by rapid inflation of the balloon with 30cc of air to note any descent in the resting pressure.	"High" in patients without soiling (n=7).<br>"Low" in two patients with soiling.<br>Not further reported.	No patients demonstrated increase in pressure on squeezing. Not further reported.	100%<br>"Weak" RAIR
**Emblem** [40]	Anal canal resting pressure<br>Assessment not further defined.	Anal canal squeeze pressure<br>Increase in pressure during squeezing.	Not defined.	Dilatation / cutback:<br>49 ± 17<br>Transposition / anoplasty:<br>47 ± 13<br>Controls: 69 ± 7	Dilatation / cutback:<br>88 ± 49<br>Transposition / anoplasty:<br>40 ± 20<br>Controls: 142 ± 55	100%
**Emblem** [45]	Maximal anal canal resting pressure<br>Assessment not further defined.	Maximal anal canal squeeze pressure<br>Assessment not further defined.	-	High/intermediate<br>***M***: 21.3 (13.9 – 29.4); ***F***: 25.0 (17.7 – 33.1).<br>Low<br>***M***: 55.9 (48.6 – 64.0); ***F***:41.2 (33.8 – 48.6).<br>Controls:<br>***M***: 67.7 (59.6 – 75.8); ***F***: 70.6 (62.5 – 78.7).<br>Expressed as mean (95% CI).	High/intermediate<br>***M***: 44.1 (15.4 – 72.1); ***F***: 21.3 (7.4 – 49.3).<br>Low<br>***M***: 105.2 (76.5 – 133.1); ***F***:44.9 (18.4 – 72.1).<br>Controls:<br>***M***: 157.4 (127.3 – 188.3); ***F***: 101.5 (71.3 – 131.7).<br>Expressed as mean (95% CI).	-
**Fukata** [41]	Anal resting pressure<br>Assessment not further defined.	-	Not defined.	High<br>*IAS seen:* 13.8 (8.1 – 17.6)<br>IAS not seen: not reported<br>Intermediate<br>*IAS seen:* 16.2 (single patient)<br>*IAS not seen:* 19.3 (10.3 – 33.8)	-	7%

First author	Resting pressure	Squeeze pressure	RAIR	Resting pressure (mmHg)	Squeeze pressure *(mmHg)*	RAIR (% present)
**Hedlund** [46]	Average anal resting tone (ART)	Maximal anal squeeze pressure (ASP).	**Rectoanal inhibition reflex:** anal response to rectal distension, performed at 14.7 and 29.4mmHg.	Average ART reduced by ~50% compared with controls.	Mean maximal ASP reduced by ~40% compared with controls.	30%
**Heikenen** [74]	**Resting internal anal sphincter pressure**. Pressure at the trough of the wave during catheter retraction through the anal canal.<br>Normal: 45- 90mmHg.	Maximal squeeze pressure. Assessment not further defined.<br>Normal values not defined.	Rectosphincteric relaxation.<br>Decrease >5mmHg below resting IAS pressure with rectal distension.	Mean: 19.5mmHg<br>Subnormal in 6/10 (60%)	Normal: 1/5 (not further reported).	60%
**Hettiarachchi** [47]	-	-	**Rectoanal reflex**: individual inflation of the rectal balloon produces a sharp spike, followed by a simple wave of rectal peristalsis and a progressive fall in IAS pressure, with complete inhibition at 100 to 150mL.	-	-	-
**Huang** [20]	Resting rectal pressure Assessment not further defined.	-	Not defined.	Modified PSARP: 50.8±1.1<br>Transperineal anal transposition: 52.2±1.3	-	0%
**Husberg** [26]	**Anal pressure.** Not further defined.	-	**Rectoanal inhibition reflex** (RAI): manometrical expression of a functional internal sphincter that affects the major part of anal resting tone. Not further defined.	**Swedish cohort:** 49±6.6 (with RAI); 38±12 (without RAI).<br>**Finnish cohort:** 31±7.2 (with RAI); 24±3.6 (without RAI).	-	74%

First author	Resting pressure	Squeeze pressure	RAIR	Resting pressure (mmHg)	Squeeze pressure *(mmHg)*	RAIR (% present)
**Ishihara** [48]	**Maximum anal canal static pressure**: assessed using station pull-through method 1-5cm from anal verge. Not further defined.	-	-	Maximum static pressure, expressed as percent of control group (by functional status).<br>**Translevator**: 55±15.3 (normal).<br>**Intermediate**: 36.4±6.4 (normal); 39.4±5.7 (constipation); 28.4±1.6 (FI).<br>**Supralevator**: 37.6±9.4 (normal); 43.2±12.3 (constipation); 28±10.9 (FI).	-	-
**Iwai** [65]	Anal resting pressure: not further defined.	Voluntary contraction pressure: not further defined.	**Anorectal reflex:** not further defined.	Pre biofeedback: 17.5±2.7<br>Post biofeedback: 19.3±1.8	Pre biofeedback: 28.1±5.7<br>Post biofeedback: 59.7±8.3	0%
**Iwai** [66]	Anal resting pressure: not further defined.	Voluntary contraction pressure: not further defined.	**Anorectal reflex:** not further defined.	With respect to Dai-Kenchu-To treatment:<br>**Pre**: 15.3±6.2<br>**Post**: 16.9±4.6	With respect to Dai-Kenchu-To treatment:<br>**Pre**: 29.4±10.4<br>**Post**: 42.5±31.5	0%
**Iwai** [68]	Anal resting pressure: not further defined.	**Voluntary contraction pressure**: measured 1cm and 2cm from the anal verge; not further defined.	**Anorectal reflex:** not further defined.	Pre biofeedback: 13.5±1.9<br>Post biofeedback: 17.9±2.0	Pre biofeedback: 14.4±2.3<br>Post biofeedback: 38.2±6.6	0%
**Iwai** [15]	-	**Voluntary anal contraction pressure**: measured 1cm and 2cm from the anal verge; mean pressure was expressed as voluntary contraction pressure.	-	-	Low:<br>- 2cm: 19.9 ± (-)<br>- 1cm: 22.1 ± (-)<br>Intermediate:<br>- 2cm: 31.5 ± 5.2<br>- 1cm: 28.8 ± 4.4<br>High:<br>- 2cm: 24.6 ± 4.9<br>- 1cm: 21.3 ± 4.2	-
**Keshtgar** [69]	Resting anal sphincter pressure: <30mmHg “poor”	-	**Rectoanal inhibitory reflex**: “good” if present on balloon inflation to 60mL. Not further defined.	Not reported.	-	Not reported.

First author	Resting pressure	Squeeze pressure	RAIR	Resting pressure (mmHg)	Squeeze pressure *(mmHg)*	RAIR (% present)
**Keshtgar** [49][5]	Resting anal sphincter pressure: <30mmHg "poor"	-	**Rectoanal inhibitory reflex**: inhibitory relaxation of the IAS (anal resting pressure reduction of > 5 mmHg)	*Low*: median 44 (31 – 72)<br>*High:* median 24 (9 – 45)	-	Low: 80%
**Kimura** [27]	Maximal anal resting pressure: not further defined.	-	**Anorectal reflex**: not further defined.	**Laparoscopic**: 25.0 ± 6.6<br>**Open**: 22.8 ± 10.3	-	**Laparoscopic**: 23%<br>**Open**: 7%
**Kudou** [28]	Resting pressure of anal internal sphincter: not further defined.	-	**Rectoanal relaxation reflex:** not further defined.	**LAARP**: 22.8 ± 8.1<br>**PSARP**: 24.3 ± 7.4	-	**LAARP**: 62%<br>**PSARP**: 29%
**Kumar** [57]	**Resting pressure of the anal canal**: calculated as the mean of four channels, with each channel RP calculated as the mean of three pressure segments.	-	**Rectoanal inhibitory reflex (RAIR):** anal sphincter pressure drop of >5mmHg. Attempts ceased if no RAIR demonstrated at 5x expected volume for age.	Infants:<br>- 17 ± 7.7 (high)<br>- 34 ± 8.6 (low)<br>- 42.43 ± 8.19 (controls)<br>Children:<br>- 21 ± 9.5 (high)<br>- 26 ± 9.9 (low)<br>- 43.43 ± 8.8 (controls)	-	Patients: 50%<br>Controls: 100%
**Langemeijer** [29]	-	**Squeeze pressure**: contraction of pelvic floor muscles with catheter tip 1 - 3cm from the anus.<br>Good: sustained squeeze >30cmH_2O.	**Inhibitory reflex**: not further defined.	-	Good: 16/24	2.5%
**Leung** [70]	-	Anal sphincter squeeze pressure: not further defined.	-	-	With respect to training program:<br>-Before: 55.5<br>-*After*: 85.4	-
**Lin** [30]	Resting anal pressure: not further defined.	-	**Rectoanal relaxation reflex** (RAR): pressure decrease of more than half the resting anal pressure following balloon inflation.	PSARP<br><4 years<br>**-**Blind pouch:25.3 ± 2.2<br>-Fistula:25.0 ± 4.8<br>>4 years<br>-*Bling pouch*: 25.7 ± 3.0<br>-*Fistula*: 26.8 ± 4.0<br><br>**R-APSA**: 26.3 ± 2.9	-	PSARP<br>-***Group 1*** (internal sphincter-saving): 57.1%<br>-***Group 2*** (incomplete internal sphincter-saving): 77.8%<br><br>*Group 3* (R-APSA): 75%

[5] "High" anomaly group excluded, due to inclusion of patients more than 18 years of age.

First author	Resting pressure	Squeeze pressure	RAIR	Resting pressure (mmHg)	Squeeze pressure *(mmHg)*	RAIR (% present)
**Lin** [31]	**Resting sphincteric pressure**: measured 0.5cm from anal verge for 1 minute.	-	**Rectoanal relaxation reflex (RAR)**: pressure drop exceeding half of the resting rectal pressure.	Resting sphincteric pressure<br>**LAR**: 15.8 ± 3.5<br>**PSARP**: 18.7 ± 4.6	-	**LAR**: 88.9%<br>**PSARP**: 30.8%
**Liu** [32]	**Anal resting pressure**: not further defined.	Anal squeezing pressure: not further defined.	**Rectoanal inhibitory reflex**: examined using "balloon method". Not further defined.	Group 1<br>Excellent/good: 33.8 ± 8.2<br>*Fair / poor*: 25.6 ± 7.4<br>Group 2<br>Excellent/good: 35.2 ± 9.7<br>*Fair / poor*: 23.2 ± 7.3	Group 1<br>Excellent/good: 60.8 ± 10.0<br>*Fair / poor*: 42.4 ± 11.6<br>Group 2<br>Excellent/good: 62.7 ± 10.7<br>*Fair / poor*: 6.3 ± 13.5	**Group 1**: 91.7%<br>**Group 2**: 87.7%
**Martins** [50]	Initial resting pressure: not further defined.	Voluntary sphincter contraction: not further defined.	**Sphincter-rectal reflexes:** considered positive when a "clear" pressure decrease in the sphincter balloon was observed on rectal balloon filling	Continent: 13.92<br>Partially continent: 10.86<br>Incontinent: 6.57<br><br>*Measure of central tendency not described.*	Continent: 22.85<br>Partially continent: 17.29<br>Incontinent: 9.86<br><br>*Measure of central tendency not described.*	0%
**Mert** [55]	**Resting pressure of the anal canal:** not further defined.	**Squeeze pressure:** not further defined.<br>**Maximum voluntary squeeze pressure:** not further defined.	**Rectoanal inhibitory reflex:** not further defined.	Mean: 45	Mean: 85	100%
**Mollard** [75]	-	-	**Rectoanal inhibitory reflex threshold** (RAIRT): distension volume to elicit a relaxation >1kPa.	-	-	Intermediate: 50%<br>**High**: 42.9%
**Nagashima** [62]	**Maximum anal pressure**: measured by withdrawing probe to anal verge.	-	-	**High**: 29.8 ± 2.1<br>**Intermediate**: 26.3 ± 5.1<br>**Low**: 37.4 ± 2.9	-	-
**Niedzielski** [33]	Resting rectal pressure (RrP): not further defined.<br>Resting anal pressure (ArP): not further defined.	-	Rectoanal inhibitory reflex: not further defined.	ArP: 11.6<br>*-Low*: 12.4<br>*-High*: 8.3	-	Total: 78%<br>- Low: 86.8%<br>- High: 52.2%
**Ninan** [71]	-	Voluntary squeeze pressure:<br>Good: >58.9<br>Moderate: 18.4 – 58.9<br>Poor: <18.4	-	-	**Good**: 6 patients<br>**Moderate**: 4 patients<br>**Poor**: 3 patients	-

First author	Resting pressure	Squeeze pressure	RAIR	Resting pressure (mmHg)	Squeeze pressure *(mmHg)*	RAIR (% present)
**Okada** [21]	Rectal pressure and maximum anal canal pressure	-	**Anorectal reflex:** not further defined.	Pre redo<br>Rectal pressure: 8.5 ± 5.0<br>Anal pressure: 16.7 ± 4.3<br>Post redo<br>Rectal pressure: 6.6 ± 1.2<br>Anal pressure: 22.7 ± 9.2	-	33%[6]
**Penninckx** [16]	Maximal anal basal pressure: not further defined.	-	Rectoanal inhibitory reflex: not further defined.	Vaginal anal canal: 51 ± 6<br>Urethral anal canal: 35 ± 11<br>Vesical anal canal: 32 ± 2<br>No orifice: 39 ± 19	-	100%
**Ray** [76]	**Basal pressure:** not further defined.	Maximum squeeze pressure: not further defined.	Rectoanal inhibitory reflex: not further defined.	**High**: range 10 – 15<br>**Intermediate**: range 30 - 50	**High**: not elicited<br>**Intermediate**: 2.5 to 3 times basal pressure	**High**: 0%<br>**Intermediate**: 66.7% (male), 76.7% (female).
**Ren** [34]	Anal canal resting pressure (ACRP)	-	Rectal anal inhibitory reflex: not further defined.	**SILAARP**:33.35 ± 12.95<br>**PSARP**: 23.06 ± 8.40	-	**SILAARP**: 86.7%<br>**PSARP**: 100%
**Rintala** [35]	**Anal basal pressure**: mean value of the three highest recordings of the anorectal pressure profile during constant pull-through.<br>**Rectal pressure**: measured for 30s 5-10cm from the anal verge, depending upon patient age.	-	**Rectoanal inhibitory reflex**: volume of the rectal balloon that produced a relaxation reflex considered the RAIR threshold volume.	Mean basal anal canal pressure:<br>**Group 1**: 27.95 ± 8.09<br>**Group 2**: 18.39 ± 5.15	-	**Group 1**: 78.6%<br>Group 2: 0%
**Rintala** [36]	**Anal resting pressure**: not further defined.	-	**Rectoanal inhibitory reflex**: >25% decrease in resting anal canal pressure, reproducible during 3 consecutive rectal stimulation.	With internal sphincter: 35.5 ± 7.4<br>Without internal sphincter: 23.5 ± 3.7	-	82%
**Rintala** [58]	**Anal resting pressure**: not further defined.	-	**Rectoanal inhibitory reflex**: >25% decrease in resting anal canal pressure, reproducible during 3 consecutive rectal stimulation.	With internal sphincter: 31.9 ± 6.7<br>Without internal sphincter: 24.4 ± 3.5	-	83%
**Rintala** [17]	Maximal anal canal pressure: not further defined.	-	Rectoanal inhibitory reflex: not further defined.	Mean: 25.8	-	90%

[6] Anorectal reflex (not further defined) was identified in 33% of the cohort both pre- and post-operatively.

First author	Resting pressure	Squeeze pressure	RAIR	Resting pressure (mmHg)	Squeeze pressure *(mmHg)*	RAIR (% present)
**Rintala** [59]	**Anal resting pressure**: not further defined.	-	**Rectoanal inhibitory reflex**: volume of the balloon that produced the relaxation reflex recorded as the threshold volume. Not further defined.	By functional outcome:<br>- Excellent: 100%<br>- ***Good***: 75%<br>- Fair – poor: 57%	-	By functional outcome:<br>- Excellent: 100%<br>- ***Good***: 75%<br>- Fair – poor: 57%
**Rintala** [60]	**Basal anal canal pressure:** not further defined. As per referenced protocol:<br>mean value of the three highest recordings of the anorectal pressure profile during constant pull-through [35].	**Maximal sphincter squeeze**: not further defined. Not defined in referenced protocol [35].	**Rectoanal inhibitory reflex**: not further defined. As per referenced protocol: volume of the rectal balloon that produced a relaxation reflex considered the RAIR threshold volume [35].	**Pre-operative** (redo): 17.7 ± 3.7<br>Post-operative: 22.8 ± 6.6	**Pre-operative** (redo): 38.2 ± 12.5<br>Post-operative: 58.9 ± 14.7	0%
**Ruttenstock** [18]	**Resting pressure**: three of four channels in the anal canal selected at 3 different levels and the mean of these readings labelled the RP.	-	**Rectoanal inhibitory reflex**: anal sphincter pressure drop of at least 10mmHg.	**Post-operative**: 62.11 ± 19.23	-	100%
**Sangkhathat** [63]	Average resting pressure in anus (ArP) and rectum (RrP): mean, not further defined.	**Peak squeeze pressure (PSP):** highest rectal pressure recorded when an inflated rectal balloon was rapidly pulled.	**Rectoanal inhibitory reflex**: reduction of anal pressure to 50% of resting pressure, in response to rectal balloon distension.	**Average RrP**: mean 5.8 (range 0.8 – 26)<br>**Average ArP**: mean 26.5 (range 5.9 – 85)	**PSP**: mean 86.3 (range 23 – 240.2)	**Total**: 66.7%<br>Constipation: 12.5%<br>Without constipation: 93.75%
**Schuster** [19]	**Maximal mean segmental pressure** (at rest): peak value of all mean pressure values calculated from the eight sampled values at different levels of the anal canal during catheter withdrawal.	-	Rectoanal inhibitory reflex: not further defined.	Mean: 75.75 ± 16.8 (46 – 85)	-	Not reported.
**Schuster** [42]	Maximal mean segmental pressure (at rest)<br>Assessment not further defined.	Maximal mean segmental pressure (during squeeze)<br>Assessment not further defined.	-	Range: 6 – 65<br><br>Measure of central tendency not provided.	Range: 21 – 102<br><br>Measure of central tendency not provided.	-

First author	Resting pressure	Squeeze pressure	RAIR	Resting pressure (mmHg)	Squeeze pressure *(mmHg)*	RAIR (% present)
**Senel** [51]	Average anal resting pressure (aARP): not further defined.	-	**Rectoanal inhibitory reflex**: >50% reduction of anal resting pressure following rectal balloon distension.	Anorectal malformation type:<br>***Intermediate***: 42.8 ± 5.9<br>***High***: 29.5 ± 12.8<br><br>Continence outcome:<br>***Good***: 42.6 ± 6.3<br>***Fair / poor***: 23.5 ± 9.4	-	94.4%
**Sonnino** [37]	-	**Maximal pressure**: normal value for continence >50mmHg.	**Rectoanal inhibitory reflex**: decrease in pressure at the level of the internal sphincter with inflation of the rectal balloon; represented graphically by a relaxation wave.	-	Pre-op (gracilis transposition; listed per patient): <10, 0, 20 – 30, 0, 20 – 40.<br><br>Post-op (listed per patient): 80, 60-80, 55, 55, 70.	Not reported.
**Tang** [52]	**Resting pressure of high-pressure zone**; reported as maximum/minimum. No reported comparison with norms.	Squeeze pressure of high-pressure zone; reported as maximum/minimum.<br>No reported comparison with norms.	-	Summary statistics not provided.<br><br>Maximal resting pressure range: 85 - 113	Summary statistics not provided.<br><br>Maximal squeeze pressure range: 73 – 238.	-
**Tong** [38]	**Resting anal pressure**: the highest mean anal pressure segments at rest.	**Squeeze anal pressure**: the highest mean anal pressure segments during voluntary squeeze.	Rectoanal relaxation reflex / rectoanal inhibitory reflex: not further defined.	**LAARPT**: 25.5 ± 8.1<br>**PSARP**: 21.8 ± 9.6]	**LAARPT**: 55.2 ± 17.3<br>**PSARP**: 50.3 ± 18.9	**LAARPT**: 84.8%<br>**PSARP**: 85.7%
**Vital Junior** [53]	**Resting pressure**: not further defined.	**Pressure response to voluntary contraction (VCP):** not further defined.	**Rectosphincteric reflex** (RSR): not further defined.	**Continent**: 30.7 ± 9.9<br>Partially continent: 23.0 ± 6.8<br>**Incontinent**: 14.7 ± 5.2	**Continent**: 65.4 ± 18.7<br>Partially continent: 55.8 ± 16.0<br>**Incontinent**: 46.6 ± 14.3	17.1%
**Wang** [54]	Rest pressure of the anus: not further defined.	-	Rectoanal inhibitory reflex: not further defined.	-	-	95.7%
**Yang** [39]	**Anal canal resting pressure** (ACRP): not further defined.	-	Rectal anal inhibitory reflex: not further defined.	**LAARPT**: 29.4 ± 7.2<br>**PSARP**: 23.4 ± 6.5	-	**LAARPT**: 81.8%<br>**PSARP**: 83.3%

aARP: average anal resting pressure**; aASP:** average anal squeeze pressure; **ACRP:** anal canal resting pressure; **ArP**: anal resting pressure**; ART**: anal resting tone; **ASP**: anal squeeze pressure; **F:** female; **FI:** fecal incontinence; **IAS**: internal anal sphincter; **LAARP:** laparoscopy-assisted anorectoplasty; **LAARPT:** laparoscopically assisted anorectal pullthrough**; LAR:** laparoscopically assisted pull-through anorectoplasty**; LSARP**: limited sagittal anorectoplasty; **M**: male; **PSARP**: posterior sagittal anorectoplasty; **PSP**: peak squeeze pressure**; RAI**: rectoanal inhibition reflex; **RAIR:** rectoanal inhibitory reflex; **RAIRT:** rectoanal inhibitory reflex threshold; **R-**

APSA: Rehbein’s mucosa-stripping endorectal pull-through**; RAR:** rectoanal relaxation reflex**; RP:** resting pressure; **RrP**: resting rectal pressure; **SILAARP**: single-incision laparoscopic-assisted anorectoplasty; **VCP:** voluntary contraction pressure; **– :** not reported.

Table S3: Preferred Reporting Items for Systematic Reviews and Meta-Analyses (PRISMA) Checklist [10].

Section / Topic	#	Checklist item	Page
Title			
Title	1	Identify the report as a systematic review, meta-analysis, or both.	1
Abstract			
Structured summary	2	Provide a structured summary including, as applicable: background; objectives; data sources; study eligibility criteria, participants, and interventions; study appraisal and synthesis methods; results; limitations; conclusions and implications of key findings; systematic review registration number.	3
Introduction			
Rationale	3	Describe the rationale for the review in the context of what is already known.	4
Objectives	4	Provide an explicit statement of questions being addressed with reference to participants, interventions, comparisons, outcomes, and study design (PICOS).	4
Methods			
Protocol and registration	5	Indicate if a review protocol exists, if and where it can be accessed (e.g., Web address), and, if available, provide registration information including registration number.	5
Eligibility criteria	6	Specify study characteristics (e.g., PICOS, length of follow-up) and report characteristics (e.g., years considered, language, publication status) used as criteria for eligibility, giving rationale.	5
Information sources	7	Describe all information sources (e.g., databases with dates of coverage, contact with study authors to identify additional studies) in the search and date last searched.	5
Search	8	Present full electronic search strategy for at least one database, including any limits used, such that it could be repeated.	48 (Table A1/2)
Study selection	9	State the process for selecting studies (i.e., screening, eligibility, included in systematic review, and, if applicable, included in the meta-analysis).	5
Data collection process	10	Describe method of data extraction from reports (e.g., piloted forms, independently, in duplicate) and any processes for obtaining and confirming data from investigators.	5
Data items	11	List and define all variables for which data were sought (e.g., PICOS, funding sources) and any assumptions and simplifications made.	5
Risk of bias in individual studies	12	Describe methods used for assessing risk of bias of individual studies (including specification of whether this was done at the study or outcome level), and how this information is to be used in any data synthesis.	6
Summary of measures	13	State the principal summary measures (e.g., risk ratio, difference in means).	-
Synthesis of results	14	Describe the methods of handling data and combining results of studies, if done, including measures of consistency (e.g., I^2) for each meta-analysis.	-
Risk of bias across studies	15	Specify any assessment of risk of bias that may affect the cumulative evidence (e.g., publication bias, selective reporting within studies).	-
Additional analyses	16	Describe methods of additional analyses (e.g., sensitivity or subgroup analyses, meta-regression), if done, indicating which were pre-specified.	-
Results			
Study selection	17	Give numbers of studies screened, assessed for eligibility, and included in the review, with reasons for exclusions at each stage, ideally with a flow diagram.	7
Study characteristics	18	For each study, present characteristics for which data were extracted (e.g., study size, PICOS, follow-up period) and provide the citations.	9
Risk of bias within studies	19	Present data on risk of bias of each study and, if available, any outcome level assessment (see item 12).	8
Results of individual studies	20	For all outcomes considered (benefits or harms), present, for each study: (a) simple summary data for each intervention group (b) effect estimates and confidence intervals, ideally with a forest plot.	27
Synthesis of results	21	Present results of each meta-analysis done, including confidence intervals and measures of consistency.	-
Risk of bias across studies	22	Present results of any assessment of risk of bias across studies (see Item 15).	45
Additional analysis	23	Give results of additional analyses, if done (e.g., sensitivity or subgroup analyses, meta-regression [see Item 16]).	-
Discussion			
Summary of evidence	24	Summarize the main findings including the strength of evidence for each main outcome; consider their relevance to key groups (e.g., healthcare providers, users, and policy makers).	41

Limitations	25	Discuss limitations at study and outcome level (e.g., risk of bias), and at review-level (e.g., incomplete retrieval of identified research, reporting bias).	45
Conclusions	26	Provide a general interpretation of the results in the context of other evidence, and implications for future research.	46
Funding			
Funding	27	Describe sources of funding for the systematic review and other support (e.g., supply of data); role of funders for the systematic review.	47
